# Neurofilament light chain predicts future dementia risk in cerebral small vessel disease

**DOI:** 10.1136/jnnp-2020-325681

**Published:** 2021-02-08

**Authors:** Marco Egle, Laurence Loubiere, Aleksandra Maceski, Jens Kuhle, Nils Peters, Hugh S Markus

**Affiliations:** 1 Stroke Research Group, Department of Clinical Neurosciences, University of Cambridge, Cambridge, UK; 2 Neurologic Clinic and Policlinic, Departments of Medicine, Biomedicine and Clinical Research, University Hospital and University of Basel, Basel, Switzerland; 3 Stroke Center, Klinik Hirslanden, Zürich, Switzerland; 4 Stroke Center and Department of Neurology, University Hospital and University of Basel, Basel, Switzerland

## Abstract

**Objectives:**

Serum neurofilament light chain (NfL) has been proposed as prognostic markers in neurogenerative disease. A cross-sectional study in cerebral small vessel disease (SVD) reported an association with cognition and disability. If NfL is to be used to predict outcome, studies are required to demonstrate baseline NfL predicts future dementia risk. Furthermore, if it is to be used as a surrogate marker in clinical trials, change in NfL over time periods typical of a clinical trial must be linked to clinical progression. In a longitudinal study of patients with lacunar stroke and confluent white matter hyperintensities, we determined whether both baseline, and change, in NfL levels were linked to changes in MRI markers, cognitive decline and dementia risk.

**Methods:**

Patients underwent MRI, cognitive testing and blood taking at baseline and annually for 3 years. Clinical and cognitive follow-up continued for 5 years.

**Results:**

NfL data were available for 113 subjects for baseline analysis, and 90 patients for the longitudinal analysis. Baseline NfL predicted cognitive decline (global cognition β*=*−0.335, SE=0.094, p=0.001) and risk of converting to dementia (HR=1.676 (95% CI 1.183 to 2.373), p=0.004). In contrast to imaging, there was no change in NfL values over the follow-up period.

**Conclusions:**

Baseline NfL predicts changes in MRI markers, cognitive decline and dementia rate over a 5 years follow-up period in SVD, suggesting NfL may be a useful prognostic marker. However, change in NfL values was not detected, and therefore NfL may not be a useful surrogate marker in clinical trials in SVD.

## Introduction

Cerebral small vessel disease (SVD) is a highly prevalent condition, which causes lacunar stroke, vascular cognitive impairment and vascular dementia.^1^ Few treatments have been shown to delay disease progression. One challenge in testing therapies is that, while on a population basis MRI features of SVD predict dementia, predicting which individual patients will progress is difficult.^2^ This has led to calls for better markers to predict progression to dementia. Furthermore such markers may be useful as surrogate markers in disease studies. Due to the variable progression rate large sample sizes are required for clinical trials, and markers, which are sensitive to change would allow treatments to be tested in smaller phase 2 trials.^3^ MRI markers including white matter hyperintensities (WMH) and diffusion tensor imaging (DTI) have been suggested as surrogate markers.^3^

Neurofilament light chain (NfL) is a blood marker sensitive to neuroaxonal damage and increased levels occur in neurodegenerative diseases.^4 5^ In a cross-sectional study in SVD, NfL levels were associated with cognition and disability.^6^ However, if NfL is to be used to predict outcome, studies are required to demonstrate baseline NfL predicts future dementia risk. Furthermore if it is to be used as a surrogate marker in clinical trials, a change in NfL level within a 2–3 years period typical of a clinical trial must be shown to be associated with clinical progression. In a longitudinal prospective study of patients with moderate-to-severe SVD, we determined whether both baseline, and change, in NfL levels predicted future cognitive decline and dementia risk.

## Methods

### Subjects

Both baseline and follow-up data were used from the St George’s Cognition and Neuroimaging in Stroke (SCANS) prospective study. A total of 121 patients with symptomatic SVD, defined as a clinical lacunar stroke syndrome with MRI evidence of an anatomically corresponding lacunar infarct, and with confluent regions of WMH graded ≥2 on the modified Fazekas scale^7^ were enroled from three stroke services in South London (St George’s Hospital, King’s College Hospital and St Thomas’ Hospital) at least 3 months post stroke.^8^ Exclusion criteria were any stroke not caused by SVD, other central nervous system diseases, major psychiatric disorders and any cause of white matter (WM) disease other than SVD. Written informed consent was obtained from all patients participating in the study. The study was granted ethical approval by Wandsworth Research Ethic Committee.

Subjects were followed up annually for 5 years with six incidences of strokes being recorded (four lacunar strokes, two intracerebral haemorrhages). MRI was performed at baseline and after 1, 2 and 3 years. Information on dementia incidence was acquired annually. All blood samples had been collected annually at the time of MRI imaging. Of the 121 subjects recruited, blood was available for 113, and in 90 patients blood samples were available from at least at two time points. Due to a small sample size, observations coming from follow-up time point 4 and 5 were removed.

### Serum NfL

All assays were performed at the same time in 2019–2020. The analysis was the same for all samples using the same single-molecule array instrument (Simoa HD-1, Quanterix, Lexington, Massachusetts, USA) in Basel. We used the capture monoclonal antibody (mAB) 47:3 and the biotinylated detector mAB 2:1 (UmanDiagnostics, Umeå, Sweden),^9^ transferred onto the Simoa platform. Bovine lyophilised NfL was acquired from UmanDiagnostics. The range of the calibrators was between 0 and 2000 pg /mL. Intra-assay and interassay variabilities were below 20%. The analytical sensitivity was 0.32 pg /mL. All samples had signals above the assay’s analytical sensitivity. The NfL analysis was blind to the dementia outcome measures.

### MRI acquisition

Images were obtained using a 1.5 T General Electric Signa HDxt MRI system^8^ employing the same image acquisition protocol at every time point. The following scan sequences referring to whole head coverage were acquired:

1. Axial Fluid Attenuated Inversion Recovery (FLAIR): repetition time (TR)=9000 ms, echo to time (TE)=130 ms, inversion time=2200 ms, 28 slices of 5 mm thickness without slice gap, field of view=240×240 mm^2^, matrix=256×192
2. Coronal T1-weighted spoiled gradient recalled echo: TR=11.5 ms, TE=5 ms, 176 slices, field of view=240×240 mm^2^, matrix=256×192, flip angle=18° and characterised by 1.1 mm^3^ isotropic voxels
3. Axial single shot spin echo planar diffusion-weighted imaging: TR=15 600 ms, TE=93.4 ms, 55 slices marked by isotropic voxels of 2.5 mm^3^, field of view=240×240 mm^2^, 8 non-diffusion-weighted-images (b=0 smm^−2^). This was succeeded by diffusion-weighted volumes with diffusion gradients applied (b=1000 smm^−2^) in 25 non-collinear directions and the negative of these.

### Conventional MRI markers

Normalised brain volume (NBV) and WMH lesion volume were computed as previously described in detail.^8 10^ NBV was computed from native T1 images as an estimate of size of the brain relative to the skull size with SIENAX as part of the FMRIB software library. To obtain the WMH measure, warped T1-weighted and FLAIR images were used to create population-specific tissue probability maps (TPMs). TPMs were employed to segment native images creating tissue classes of WM, WMH, grey matter (GM) and cerebrospinal fluid (CSF). WMH lesion volume was computed by binarising the segmentations at a manually determined threshold. WMH was calculated by taking the ratio of WMH volume to the total cerebral volume, which is composed of the sum of GM, WM and WMH at a tissue probability threshold ≥0.2. Lacune count (lacune), defined as 3–15 mm in diameter, and cerebral microbleed count (CMB), defined as focal spots up to 10 mm in diameter, were detected by an independent trained rater in line with agreed neuroimaging standards.^11 12^

### DTI analysis

Two methods to assess WM ultrastructure on DTI were used:

#### All WM histogram analysis measure

The analysis has been described previously.^8^ In brief, DTI scans were preprocessed and eddy-corrected using FMRIB's Diffusion Toolbox in FSL software. Susceptibility distortions were unwarped by normalising the images to the T1 images in the phase-encoding direction. DTIFIT was then applied to create mean diffusivity maps. FLAIR to T1W and T1W to b0 registrations were performed and the affine transformation matrices were concatenated to produce a FLAIR-to-DTI transformation.^13^ The TPMs were registered into DTI space employing these transformations. A hard segmentation method was applied to generate maps of tissue classes. Histogram analysis was conducted on the MD map in all WM regions. The summary histogram measures were derived from normalised histograms with 1000 bins. The primary outcome measure was mean diffusivity peak height (MDPH) in the WM, as this has previously been shown to be the most sensitive parameter to change in the SCANS study.^8^

#### Peak width of skeletonised mean diffusivity (PSMD)

The PSMD is a fully automatically computed imaging marker being publicly available (http://psmd-marker.com) and has been fully described previously.^14^ The computation is divided into four main modules: (1) DTI sequence on MRI, (2) WM tract skeletonisation using tract-based statistics, (3) excluding CSF-prone regions employing a custom mask and (4) histogram values of mean diffusion on the skeleton. The PSMD measure refers to the difference between the 5th and 95th percentiles of the histogram distribution.

### Cognition and dementia diagnosis

Standardised tasks sensitive in capturing the impaired cognitive function in SVD were carried out annually. Full descriptions of the tasks have been published previously.^15^ Cognitive index scores were created by grouping the standardised measures into subdomain scores^15^: global cognition score (Global), executive function (EF) and processing speed (PS).

Dementia was diagnosed using the Diagnostic and Statistical Manual of Mental Disorders-5 (DSM-5) definition of major neurocognitive disorder, as previously described,^16^ and was made under one of the following criteria:

1. Dementia was diagnosed in a memory clinic or in a similar clinical service
2. A neurologist and a clinical neuropsychologists who were both blind to all imaging and risk factor information reviewed medical records as well as cognitive data and together agreed that a patient met the DSM-5 criteria.
3. Significant cognitive impairment and reduced capabilities in daily living indicated by a consistently reduced score of <24 in the mini-mental state examination (MMSE) and of ≤7 on the instrumental activities of daily living.

### Disability

Disability was assessed using the modified Rankin Scale,^17 18^ a 7-point scale measuring disability during stroke.

### Statistical analysis

The statistical analysis was carried out using R (version 3.6.3). NfL, WMH lesion load, lacune and CMB count were log 10 transformed due to the skewness of their data distribution. One outlier observation at baseline was excluded as being nearly 30 times higher than any of the patient’s follow-up NfL values. This patient did not show any clinical aspects that could explain such a high value as being 63 years old, having been diagnosed with diabetes and having suffered a clinical stroke 7 years ago.

Associations between NfL and DTI-MRI variables were tested using Spearman’s rank correlations. Relationships between NfL or imaging measures and cognition were tested using linear regression after controlling for the clinical markers age, gender and premorbid IQ (NART). Employing a Poisson regression model the association between NfL and disability was tested while controlling for baseline age.

The effect of time on change in cognition for Global and PS over the five follow-up years was estimated using a linear mixed model (lme4).^19^ Fixed effect variation was explained by the study’s duration, and random effect variation accounted for the residual inter-individual differences. The linear trajectory of each patient’s intercept and slope were let to vary with both fixed and random effects. The average fixed effects slopes of time are the average annualised change rate for a given cognition. Using maximum likelihood estimation to fit the model, the missing slope for one participant was estimated by taking information from all other patients included in the model.

To determine the predictive association between baseline NfL or baseline DTI and cognitive decline, a linear regression model was employed. Baseline cognition together with the clinical markers age, gender and NART were added as confounders. Employing a logistic regression, it was furthermore tested whether baseline NfL predicted an increase vs no increase in mRS as a binary outcome over 5 years while controlling for age and its baseline disability score.

A cox regression model was used to test whether the baseline NfL or imaging marker predicted dementia conversion. The proportional hazards assumption was met on the basis of Schoenfeld residuals. To assess how well the predictive models’ discriminatory ability classifies patients converting to dementia and those with no dementia at varying threshold, ROC curves were computed and the area under the curve (AUC) was estimated.^20^ Baseline NfL and baseline clinical, imaging, cognitive characteristics were compared between those patients showing more and those with less cognitive decline by employing a Welch’s t-test for variables with parametric distribution and the Wilcoxon rank sum test for variables with a non-parametric distribution.

The effect of time on change in the imaging marker and in NfL over the three follow-up years was estimated using a linear mixed model as previously described (lme4).^19^ Using maximum likelihood estimation to fit the model, the missing DTI slope for two participants was estimated by taking information from all other patients included in the model. For the dementia analysis, observations after the dementia diagnosis were omitted.

To determine the predictive association between the change in the markers and cognitive decline or dementia conversion, a linear regression or a cox regression model adjusted by age, gender and premorbid IQ was employed. The proportional hazards assumption was met on the basis of Schoenfeld residuals and the AUC was estimated.^20^ Differences in clinical characteristics and estimated changes in NfL and in the imaging markers between patients converting to dementia and those not converting to dementia/ censored over 5 years were tested using the permutation Welch’s t-test.

Finally it was also tested whether baseline and change in NfL predicted change in lacunes, CMB employing a logistic regression model controlling for the imaging marker’s baseline and age. The AUC was computed to assess diagnostic discriminatory ability in correctly classifying the binary outcome measures.^20^ The association between baseline and change in NfL and change in NBV and in WMH was tested using a linear regression model while controlling for the imaging marker’s intercept value coming from the linear mixed model and baseline age.

### Data availability

Cohort data can be shared on reasonable request for scientific purpose by contacting the corresponding author.

## Results

### Cross-sectional analysis

Study began in January 2008 and was completed in October 2013 with a consecutive case series design. Recruitment started in December 2007 and finished in August 2010. NfL data were available for 113 subjects. Clinical characteristics, cognitive measures and SVD markers at baseline are shown in table 1. NfL levels had a mean of 36.505 pg/mL, a median of 23.20 pg/mL, an IQR of 16.90 pg/mL and a minimum and maximum of 5.7 pg/mL and 708.9 pg /mL, respectively. The normal range for NfL levels using this assay has a mean of 34.69 (SD 13.09) pg/mL for age 60–70, and a mean of 45.85 (SD 15.31) pg /mL above age 70 years.^21^

**Table 1:** Baseline characteristics referring to clinical, cognitive and SVD markers

Baseline characteristics (n=113)	
Clinical characteristics	
Age, mean (SD)	70.012 (9.911)
Sex, (% male)	74 (0.66)
NART, mean (SD)	99.301 (15.544)
mRS score, median (range), IQR	1 (0–4) 2
Cognition	
Global, mean (SD)	−0.654 (0.850)
EF, mean (SD)	−0.869 (1.092)
PS, mean (SD)	−0.987 (0.910)
SVD marker	
Serum NfL (pg/mL), mean (range), median (IQR)	36.505 (5.7–708.9) 23.20 (16.90)
MDPH (mm^2^/s), mean (SD)	0.015 (0.003)
PSMD (mm^2^/s), mean (SD)	3.864e-04 (1.113e-04)
NBV (ml), mean (SD)	1292.199 (87.946)
WMH (% brain), mean (range) median (IQR)	3.507 (0.29–12.81) 3.210 (2.948)
Lacune, median (range) IQR	2 (0–27) 4
CMB, median (range) IQR	0 (0–144) 2

CMB, cerebral microbleeds count; EF, executive function; Global, global cognition; Lacune, lacune count; MDPH, mean diffusivity normalised peak height; mRS score, modified rankin scale; NART, premorbid IQ; NBV, normalised brain volume; NfL, neurofilament light chain; PS, processing speed; PSMD, Peak Width of Skeletonised Mean Diffusivity; WMH, White Matter Hyperintensity Lesion Load.

**Figure 1:**
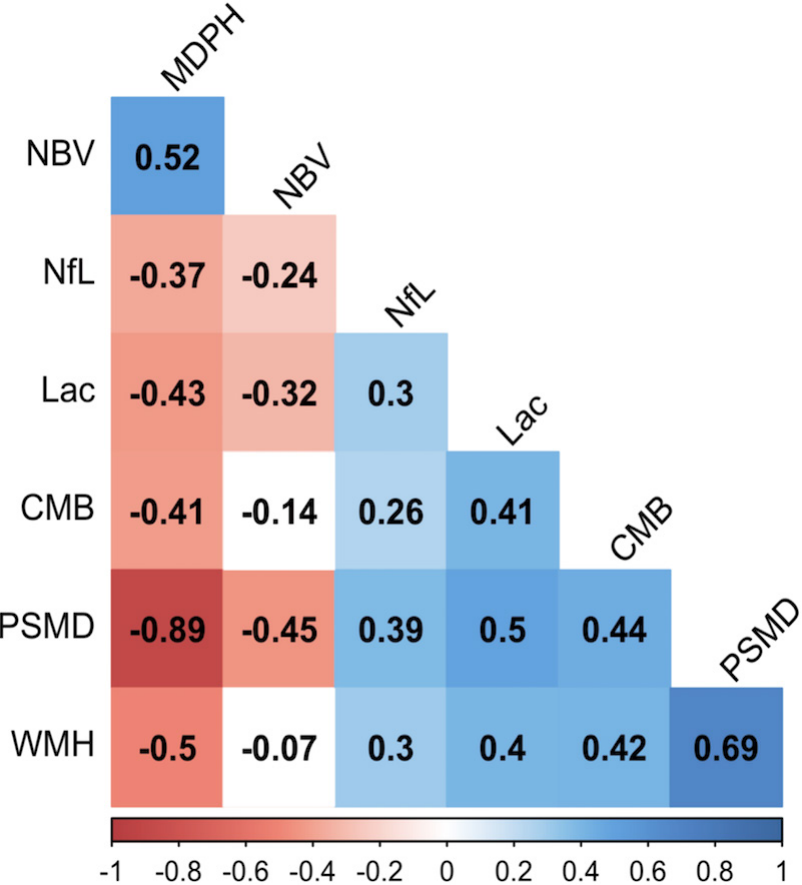
Higher levels of NfL was associated with a higher SVD marker on imaging. Spearman’s rank correlation shows the cross-sectional associations between MRI markers and NfL. Blue and red quartiles show significant positive and negative correlations. White tiles show no significant association. CMB, cerebral microbleed count; Lac, lacune count; MDPH, mean diffusivity normalised peak height; NfL, neurofilament light chain; NBV, normalised brain volume; PSMD, peak width of skeletonised mean diffusivity; SVD, small vessel disease; WMH, white matter hyperintensity.

NfL levels inversely associated with global cognitive function, the executive function and processing speed subdomain scores (table 2). Serum NfL also negatively correlated with disability (table 2). Higher NfL levels were positively associated with lacune count, CMB count, WMH and with DTI measures (higher PSMD and lower MDPH), and negatively with NBV (figure 1). We also determined whether association with cognition persisted after controlling for DTI (MDPH) and for the clinical markers; the association remained significant for PS but not for Global or EF (table 2).

**Table 2:** Cross-sectional regression between DTI and/or NfL and cognition or disability

	Regression on cognition with single marker						Regression on cognition with multiple markers						Regression on disability	
	Global		EF		PS		Global		EF		PS		mRS score	
	β (SE)	Adj. R	β (SE)	Adj. R	β (SE)	Adj. R	β (SE)	Adj. R	β (SE)	Adj. R	β (SE)	Adj. R	β (SE)	HL R^2^
Serum NfL (pg/mL) (log 10)	−0.201 (0.072), p=**0.006**	0.459	−0.155 (0.074), p=**0.038**	0.429	−0.244 (0.082), p=**0.004**	0.288	−0.118 (0.075), p=0.119	0.510	−0.086 (0.080), p=0.284	0.450	−0.187 (0.086), p=**0.031**	0.3660	0.279 (0.079), p=**0.0004**	0.1109
MDPH (mm^2^/s)	0.290 (0.071), p=**8.42e−05**	0.503	0.221 (0.075), p=**0.004**	0.449	0.330 (0.082), p=**0.0001**	0.342	0.255 (0.074), p=**0.001**		0.1951 (0.079), p=**0.015**		0.273 (0.084), p=**0.002**			
PSMD (mm^2^/s)	−0.277 (0.067), p=**7.23e−05**	0.506	−0.227 (0.070), p=**0.002**	0.462	−0.322 (0.078), p=**6.70e−05**	0.344								

Significant at p<0.05.

Values show standardised regression coefficients β and SEs for predictor variables in regression models.

Adj R^2^, adjusted explained variance; DTI, diffusion tensor imaging; EF, executive function; Global, global cognition; HL R^2^, Hosmer and Lemeshow’s R^2^; MDPH, mean diffusivity normalised peak height; mRS score, modified rankin scale; NfL, neurofilament light chain; PS, processing speed; PSMD, peak width of skeletonised mean diffusivity.

### Longitudinal analysis

#### Prediction by baseline NfL

There was a significant decline in both Global and PS over the 5 years follow-up (table 3). Higher NfL at baseline predicted lower function in Global (β*=*−0.335 (SE=0.094), p=0.001) independently of the clinical markers and baseline cognition (table 4). Higher NfL also predicted lower function in Global (β*=*−0.303 (SE=0.095), p=0.002), when controlling DTI to the existing regression model. Comparing patients showing more versus less cognitive decline further showed that there were significant baseline differences between the two groups for NART, MMSE score, NfL levels, NBV, lacune count and DTI measures but not for age, education, WMH or CMB (online supplemental table 1). Baseline NfL did not predict increases in mRS score (β=0.407 (SE=0.864), p=0.638).

10.1136/jnnp-2020-325681.supp1 Supplementary data

**Table 3:** Annualised cognitive and imaging change rate and over 5 years and 3 years, respectively

SVD markers				
Cognition (N= 90)	Estimated mean baseline value (CI)	Estimated mean annual change (CI)	t- value	P value
Global	−0.486 (−0.653 to −0.319)	−0.026 (−0.044 to −0.008)	−2.83	**0.005**
PS	−0.826 (-0.998 to −0.655)	−0.059 (−0.088 to −0.029)	−3.93	**<0.001**
Imaging characteristics (n=90)	Estimated mean baseline value (CI)	Estimated mean annual change rate (CI)	t- value	P value
Serum NfL (pg/mL) (log 10)	1.330 (1.279 to 1.381)	0.0011 (−0.017 to 0.019)	0.12	0.903
MDPH (mm^2^/s)	0.015 (0.002 to 0.016)	−0.0004 (−0.0005 to −0.0003)	−10.34	**<0.001**
PSMD (mm^2^/s)	3.744e-04 (3.544e-04 to 3.943e04)	1.316e-05 (9.467e-06 to 1.686e-05)	6.981	**<0.001**
WMH (% brain) (log 10)	0.438 (0.370 to 0.506)	0.084 (0.075 to 0.093)	17.95	**<0.001**
NBV (mL)	1294.21 (1277.007 to 1311.410)	−9.017 (−10.764 to −7.270)	−10.12	**<0.001**
	Median baseline value (range)	Number of patients with incident findings		
Lacune (count)	2 (0 to 26)	24		
CMB (count)	0 (0 to 41)	32		

Estimated annual rates of change are defined as the mean estimates of the fixed effects from the linear mixed effect models with their 95% CI.

Significant at p value <0.05.

CMB, cerebral microbleeds count; Global, global cognition; Lacune, lacune count; MDPH, mean diffusivity normalised peak height; NBV, normalised brain volume; NfL, neurofilament light chain; PS, processing speed; PSMD, peak width of skeletonised mean diffusivity; SVD, small vessel disease; WMH, white matter hyperintensity lesion load.

**Table 4:** Longitudinal analysis

	Baseline marker prediction									
	Dementia conversion (n=19)				Decline in Global			Decline in PS		
	β (SE)	P value	HR (95% CI)	AUC	β (SE)	P value	Adj. R	β (SE)	P value	Adj. R
Serum NfL (pg/mL) (log 10)	0.516 (0.178)	**0.004**	1.676 (1.183 to 2.373)	0.775	−0.335 (0.094)	**0.001**	0.353	−0.277 (0.114)	**0.017**	0.050
MDPH (mm^2^/s)	−0.848 (0.265)	**0.001**	0.428 (0.255 to 0.719)	0.791	0.148 (0.106)	0.169	0.265	0.139 (0.123)	0.262	−0.006
PSMD (mm^2^/s)	0.507 (0.155)	**0.001**	1.661 (1.225 to 2.252)	0.758	−0.215 (0.098)	**0.032**	0.290	−0.143 (0.116)	0.224	−0.003
Serum NfL (pg /mL) (log10) indep. MDPH (mm^2^/s)	0.369 (0.179)	**0.040**	1.446 (1.017 to 2.055)	0.804						
Serum NfL (pg /mL) (log10) indep. PSMD (mm^2^/s)					−0.303 (0.095)	**0.002**	0.365			

DTI and NfL baseline markers predicting cognitive decline and dementia conversion.

Values show standardised regression coefficients: β and SEs for predictor variables in regression models of dementia conversion and decline in cognition. Significant at p value <0.05.

Adj R^2^, adjusted explained variance; AUC, area under the curve; DTI, diffusion tensor imaging; Global, global cognition; MDPH, mean diffusivity normalised peak height; NfL, neurofilament light chain; PS, processing speed; PSMD, peak width of skeletonised mean diffusivity.

A total of 107 patients had complete baseline DTI and NfL data of whom 19 converted to dementia over time. Higher baseline NfL predicted the risk of converting to dementia while accounting for the clinical markers (HR=1.676 (95% CI=1.183–2.373), p=0.004, table 4). MRI parameters also predicted both cognitive decline and dementia (table 4). The AUC for prediction for dementia was 0.775 for NfL, 0.791 for MDPH and 0.758 for PSMD. To determine whether NfL contributed additional information above that provided by DTI, we additionally included baseline MDPH in the model resulting an increase in AUC of 0.804. Baseline MRI images for one patient aged 70 with a high baseline NfL level, 73.4 pg/mL, and who converted to dementia can be found in online supplemental file 1 A, B.

10.1136/jnnp-2020-325681.supp2 Supplementary data

Baseline NfL predicted changes during follow-up in the number of lacunes and CMB, and also NBV independent of their initial MRI baseline values and the patient's age (table 5). The model’s AUC was 0.773 and 0.822 for lacunes and CMB, respectively.

**Table 5:** Longitudinal analysis

	Change marker prediction									
	Dementia conversion (n=15)				Decline in Global			Decline in PS		
Cohort	β (SE)	P value	HR (95% CI)	AUC	β (SE)	P value	Adj. R	β (SE)	P value	Adj. R
Serum NfL (pg/mL) (log 10)	0.178 (0.301)	0.554	1.195 (0.663 to 2.155)	0.705	−0.051 (0.104)	0.625	0.070	0.035 (0.108)	0.751	−0.009
MDPH (mm^2^/s)	−0.813 (0.297)	**0.006**	0.444 (0.248 to 0.794)	0.788	0.260 (0.102)	**0.012**	0.134	0.049 (0.110)	0.655	−0.008
PSMD (mm^2^/s)	0.594 (0.293)	**0.043**	1.812 (1.020 to 3.219)	0.761	−0.362 (0.100)	**0.001**	0.199	−0.230 (0.108)	**0.036**	0.040

Change in DTI but not in NfL predicts cognitive decline and dementia conversion.

Values show standardised regression coefficients: β and SEs for predictor variables in regression models of dementia conversion and decline in cognition. Significant at p value <0.05.

Adj R^2^, adjusted explained variance; AUC, area under the curve; DTI, diffusion tensor imaging; Global, global cognition; MDPH, mean diffusivity normalised peak height; NfL, neurofilament light chain; PS, processing speed; PSMD, peak width of skeletonised mean diffusivity.

#### Prediction by change in NfL

**Figure 2:**
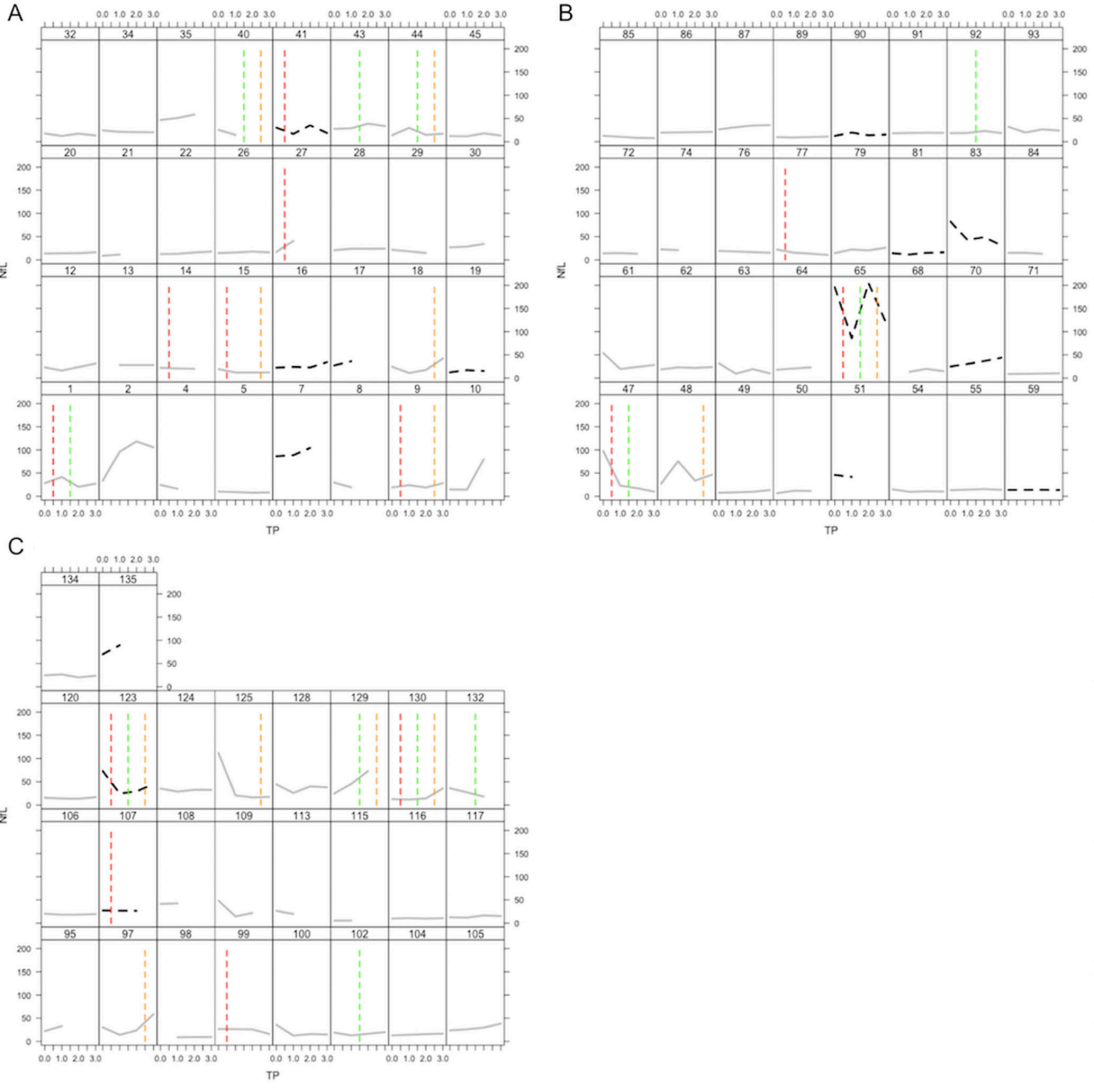
NfL measures and incidences of new lacune over time per patient. The grey line refers to NfL levels over time in patients without dementia conversion. The dotted black line refers to NfL levels over time in patients with dementia conversion. The red (left), green (middle) and orange (right) dotted vertical lines represent an incidence of a new lacune in a patient between baseline and TP 1, TP 1 and 2, TP 2 and 3, respectively. NfL, neurofilament light chain; TP, time point.

There was no significant overall change in NfL over the 3 years follow-up period (table 3). In view of this lack of change in NfL, there was no association with dementia conversion (n=15) or change in any conventional MRI measure (tables 5 and 6). The individual NfL over time per patient together with incidences in lacune are found in figure 2A–C. In contrast, DTI significantly changed over time (both assessed by MDPH and PSMD, table 3) that predicted dementia conversion (table 6). Detailed information regarding serial change of NfL, MRI markers and cognition for patients converting and not converting to dementia are found in online supplemental table 2). There were significant differences between the two patient groups for change in MDPH but not PSMD or NfL.

10.1136/jnnp-2020-325681.supp3 Supplementary data

**Table 6:** The association between baseline and change in NfL marker and change in MRI markers over 3 years

	Change in lacunes	Change in CMB	Change in WMH	Change in NBV
Serum NfL baseline (pg/mL) (log10)	0.643 (0.319), p=**0.044**, AUC=0.773	0.831 (0.320), p=**0.010**, AUC=0.822	0.107 (0.094), p=0.257	−0.234 (0.098), p=**0.019**, Adj. R^2^ *=*0.227
Serum NfL change (pg/mL)	−0.083 (0.240), p=0.730	0.313 (0.240), p=0.193	0.124 (0.108), p=0.255	0.030 (0.108), p=0.779

Values show standardised regression coefficients β and SEs for predictor variables in regression models of change in imaging marker. Significant at p<0.05.

Adj R^2^, adjusted explained variance; AUC, area under the curve; CMB, cerebral microbleeds; Lacune, lacune count; NBV, normalised brain volume; NfL, serum neurofilament light chain; WMH, white matter hyperintensity lesion load.

## Discussion

In this longitudinal prospective study in patients with moderate-to-severe SVD, NfL levels at baseline predicted both cognitive decline and conversion to dementia. The level of prediction was similar to that found for DTI, which has been previously suggested as the most sensitive marker to WM damage in SVD,^3 14^ and has been shown to predict future dementia risk.^16^ These findings suggest that NfL may be a useful marker to identify patients with SVD who are at higher risk of dementia. Previous evidence has shown that baseline NfL predicted disease severity and survival in CADASIL.^22^

In contrast, change in NfL measured on repeat testing at yearly intervals did not predict cognitive decline or dementia. This was because no significant change in NfL levels could be detected during the follow-up period. Previous evidence has shown that serum NfL proved to be a reliable marker.^23^ In contrast, change in DTI, with sampling over the same time periods as NfL levels, predicted dementia risk. This implies that while NfL levels may provide a simple marker to predict risk, they may not be useful in assessing outcome in clinical trials with a duration of a few years, where change in the surrogate marker needs to predict change in a clinical marker such as conversion to dementia.

A previous cross-sectional study has shown that NfL levels was associated with the markers of disease burden, cognition and disability in SVD.^6^ Cross-sectional analysis of our baseline data confirmed the results with NfL levels associating with the degree of cognitive impairment, of disability and markers of disease burden.

We also showed that NfL at baseline predicts changes in imaging markers of SVD burden such as NBV, CMB and lacunes but not WMH in the longitudinal analysis. This evidence is partly in line with previous findings in a subgroup (<60 years of age) of the community-based stroke-free cohort showing that baseline but also change in NfL levels were the two main determinants for explaining brain atrophy.^21^ It is also broadly consistent with recent evidence in mild SVD showing baseline NfL predicts risk of future lacunes.^24^

Why there was no significant change in NfL over 3 years in SCANS is uncertain and may be explained by dynamic changes in NfL post stroke.^25 26^ Findings from patients with small subcortical infarcts demonstrated that NfL was elevated but eventually returned to normal levels after 15 months.^26^ In SCANS blood sampling was unfortunately only taken annually not allowing us to test monthly changes in NfL levels. An alternative explanation for the lack of significant change is that the rate of change in NfL over 3 years with a sample size of 90 patients is too low in this chronic disease for any association to be detected.

This study has a number of strengths. Repeated sampling of both MRI and blood was performed and data on dementia conversion at 5 years was available for all subjects. MRI included both conventional markers and DTI, allowing the comparative performance of prediction by NfL levels with those of MRI markers to be determined. It also has limitations. NfL levels at more than one time point were not available on all subjects. It has been shown that dropouts had worse cognitive function than those who remained in the study, which might result in an underestimation of the magnitude of cognitive, MRI and NfL changes over time.^8^

This prospective cohort study demonstrates that baseline NfL values predict cognitive decline and dementia rate over a 5 years follow-up duration in patients with severe SVD. NfL may be a useful prognostic marker in this disease. However, in contrast to DTI, change in NfL values was not detected over a 3 years follow-up period with annual sampling, suggesting NfL is unlikely to be a useful surrogate marker in a phase 2 clinical trial, although further larger studies are required to confirm this finding.

## Data Availability

Data are available upon reasonable request. Cohort data can be shared upon reasonable request for scientific purpose by contacting the corresponding author. Anonymised data will be made available to qualified investigators on reasonable request to the corresponding author.
