# Supplementary material for: Neurofilament light chain predicts future dementia risk in cerebral small vessel disease
Source: J Neurol Neurosurg Psychiatry. 2021 Feb 8;92(6):582–9. doi: 10.1136/jnnp-2020-325681 (PMC8142459; doi:10.1136/jnnp-2020-325681)
Supplement: Supplementary data [file jnnp-2020-325681supp001.pdf]

Supplementary Table 1: Clinical, NfL and imaging characteristics between patients with less vs. more decline in the longitudinal sample. Differences were tested using two-sample Welch's t-test for variables with a parametric distribution and the Wilcoxon rank sum test for variables with a non-parametric distribution. There was a significant difference for NART, MMSE, NfL, NBV, Lacunes, MDPH and PSMD.

	**Less Decline (N=45)**	**More Decline (N=45)**	**Overall (N=90)**
**Age**			
Mean (SD)	68.4 (9.61)	70.9 (9.31)	69.7 (9.49)
Median [Min, Max]	69.7 [43.6, 88.8]	71.3 [46.0, 87.7]	70.8 [43.6, 88.8]
**NART ***			
Mean (SD)	105 (15.4)	97.4 (14.6)	101 (15.4)
Median [Min, Max]	106 [76.0, 127]	98.0 [75.0, 126]	103 [75.0, 127]
**Education**			
Mean (SD)	12.4 (3.70)	11.6 (3.53)	12.0 (3.61)
Median [Min, Max]	11.0 [8.50, 24.0]	11.0 [4.00, 22.0]	11.0 [4.00, 24.0]
**MMSE***			
Mean (SD)	28.9 (1.09)	27.2 (2.59)	28.0 (2.15)
Median [Min, Max]	29.0 [26.0, 30.0]	28.0 [18.0, 30.0]	29.0 [18.0, 30.0]
**NfL***			
Mean (SD)	19.2 (9.11)	37.6 (34.3)	28.5 (26.7)
Median [Min, Max]	17.9 [5.70, 48.7]	26.0 [6.60, 196]	22.2 [5.70, 196]
**NBV***			
Mean (SD)	1320 (60.9)	1270 (97.3)	1290 (84.6)
Median [Min, Max]	1310 [1230, 1500]	1270 [1060, 1500]	1300 [1060, 1500]
**WMH**			
Mean (SD)	3.06 (2.22)	3.82 (2.61)	3.44 (2.44)
Median [Min, Max]	2.65 [0.290, 10.1]	3.61 [0.460, 12.8]	3.06 [0.290, 12.8]
**Lacunes***			
Mean (SD)	2.16 (2.76)	5.69 (6.31)	3.92 (5.16)
Median [Min, Max]	1.00 [0, 11.0]	3.00 [0, 26.0]	2.00 [0, 26.0]
**CMB**			
Mean (SD)	1.96 (4.26)	3.78 (8.74)	2.87 (6.90)
Median [Min, Max]	0 [0, 21.0]	0 [0, 41.0]	0 [0, 41.0]
**MDPH***			
Mean (SD)	0.0161 (0.00274)	0.0144 (0.00224)	0.0153 (0.00263)
Median [Min, Max]	0.0155 [0.0113, 0.0225]	0.0142 [0.00816, 0.0192]	0.0149 [0.00816, 0.0225]
**PSMD***			
Mean (SD)	0.000347 (0.0000747)	0.000403 (0.000109)	0.000375 (0.0000968)
Median [Min, Max]	0.000350 [0.000231, 0.000539]	0.000382 [0.000261, 0.000790]	0.000364 [0.000231, 0.000790]

** $p < 0.05$*

NART- premorbid IQ score, MMSE- Mini–Mental State Examination, NfL- serum neurofilament light chain, NBV- normalised brain volume, WMH- white matter hyperintensity, CMB- cerebral microbleeds, MDPH- mean diffusivity normalised peak height, PSMD- peak width skeletonized mean diffusivity
