# Supplementary material for: Neurofilament light chain predicts future dementia risk in cerebral small vessel disease
Source: J Neurol Neurosurg Psychiatry. 2021 Feb 8;92(6):582–9. doi: 10.1136/jnnp-2020-325681 (PMC8142459; doi:10.1136/jnnp-2020-325681)
Supplement: Supplementary data [file jnnp-2020-325681supp002.pdf]

A)

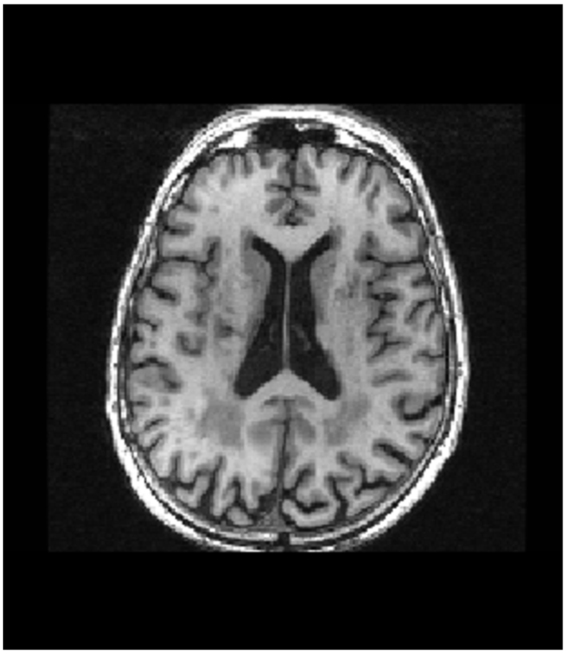

B)

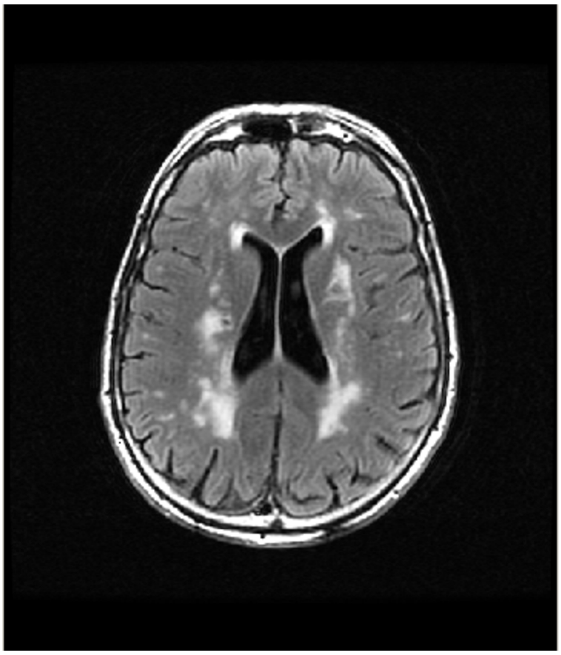

Supplementary Figure 1A-B. MRI images of a patient aged 70 with a high NfL level (73.4 pg/mL) at baseline and who later converted to dementia. The MRI sequences (A) T1 and (B) FLAIR are shown.
