# Supplementary material for: Neurofilament light chain predicts future dementia risk in cerebral small vessel disease
Source: J Neurol Neurosurg Psychiatry. 2021 Feb 8;92(6):582–9. doi: 10.1136/jnnp-2020-325681 (PMC8142459; doi:10.1136/jnnp-2020-325681)
Supplement: Supplementary data [file jnnp-2020-325681supp003.pdf]

Supplementary Table 2. Differences in clinical characteristics and estimated changes in NfL and in imaging markers between patients converting to dementia and those without dementia/ censored over 5 years. Values post dementia were removed. Permutation Welch's t-test showed that there were significant differences between the groups for Intercept NfL, Intercept MDPH, Slope MDPH.

	**Censored, no dementia (N=75)**	**Developed dementia (N=15)**	**Overall (N=90)**
**Age**			
Mean (SD)	68.9 (9.55)	73.5 (8.47)	69.7 (9.49)
Median [Min, Max]	70.6 [43.6, 88.8]	73.8 [53.8, 87.7]	70.8 [43.6, 88.8]
**NART**			
Mean (SD)	102 (15.1)	95.2 (16.1)	101 (15.4)
Median [Min, Max]	103 [76.0, 127]	90.0 [75.0, 126]	103 [75.0, 127]
**Intercept NfL***			
Mean (SD)	1.30 (0.168)	1.49 (0.268)	1.33 (0.200)
Median [Min, Max]	1.30 [0.850, 1.73]	1.45 [1.16, 2.06]	1.32 [0.850, 2.06]
**Slope NfL**			
Mean (SD)	-0.000208 (0.0315)	0.00638 (0.0186)	0.000890 (0.0297)
Median [Min, Max]	-0.00124 [-0.119, 0.0867]	0.00431 [-0.0348, 0.0406]	0.000890 [-0.119, 0.0867]
**Intercept MDPH***			
Mean (SD)	0.0156 (0.00261)	0.0136 (0.00204)	0.0153 (0.00263)
Median [Min, Max]	0.0152 [0.00979, 0.0232]	0.0135 [0.00843, 0.0165]	0.0151 [0.00843, 0.0232]
**Slope MDPH***			
Mean (SD)	-0.000377 (0.000100)	-0.000446 (0.0000769)	-0.000388 (0.0000996)
Median [Min, Max]	-0.000384 [-0.000739, -0.0000385]	-0.000408 [-0.000623, -0.000373]	-0.000393 [-0.000739, -0.0000385]
**Intercept PSMD**			
Mean (SD)	0.000366 (0.0000845)	0.000417 (0.000128)	0.000374 (0.0000944)
Median [Min, Max]	0.000358 [0.000229, 0.000659]	0.000390 [0.000291, 0.000782]	0.000362 [0.000229, 0.000782]
**Slope PSMD**			
Mean (SD)	0.0000120 (0.0000113)	0.0000174 (0.0000177)	0.0000129 (0.0000127)
Median [Min, Max]	0.0000112 [-0.0000464, 0.0000340]	0.0000138 [-0.0000274, 0.0000507]	0.0000120 [-0.0000464, 0.0000507]

* P-values < 0.05
